# Reframing the Moral Limits of Markets Debate: Social Domains, Values, Allocation Methods

**DOI:** 10.1007/s10551-016-3346-9

**Published:** 2016-10-19

**Authors:** Ben Wempe, Jeff Frooman

**Affiliations:** 1 Department of Business-Society Management, Rotterdam School of Management, Erasmus University Rotterdam, PO Box 1738, 3000 DR Rotterdam, The Netherlands; 2 Department of Philosophy, University of New Brunswick, PO Box 4400, Fredericton, NB E3B 4C4 Canada

**Keywords:** Allocation methods, Education, Healthcare, Moral limits of markets, Sphere differentiation, Values

## Abstract

What should and what should not be for sale in a society? This is the central question in the Moral Limits of Markets (MLM) debate, which is conducted by a group of business ethicists and liberal egalitarian political theorists. These MLM theorists, which we will dub ‘market moralists,’ all put forward a specific version of the argument that while the market is well suited to allocate some categories of goods and services, it is undesirable for the allocation of other such categories. We argue that the current MLM debate is too much framed in terms of a market/non-market dichotomy. Moreover, authors tend to distinguish insufficiently between values such as freedom, equality, and efficiency, and allocation methods such as the market, the queue, and rationing. We introduce a new conceptual scheme consisting of societal domains, values, and allocation methods to provide a better structure for this debate. The argument is illustrated from the education and healthcare domains.

## Introduction

The ‘expansionist tendency of the market’ (Buchanan 1985) has triggered a fierce debate on the ‘Moral Limits of Markets’ (MLM). The central question here is whether we should simply put up everything for sale, or whether there are certain goods and services that should not be allocated via the market. The most general characterization of the MLM debate delineates two broad positions. On the one hand are the market advocates who argue that the free market is the best way for society to ‘allocate scarce goods and necessary burdens’ (Elster 1992). There are two different reasons why market advocates believe this. They typically point to (1) the importance of personal liberty and the fact that letting people engage in voluntary exchanges respects their freedom; and (2) the importance of the general welfare, and the understanding that when two people freely enter into a contract, both do so on the belief that each is to gain, and thus, all other things being equal, overall welfare is to be increased.

On the other hand are the market moralists. These scholars do not accept the market as a means for solving all problems of allocation and coordination in society. Market moralists also have two core reasons for their position. They typically argue that (1) many market choices are not really free, as they are made against a background of structural inequalities, and will result in an unjust distribution of goods and services; and (2) certain goods and social practices will be corrupted or degraded if bought and sold on the market.

An example which may help bring out how these two sets of argumentation play out is price gouging. Several authors have recently argued about the moral permissibility of price gouging and the impact on civic virtue it is likely to have (e.g., Angel and McCabe 2009; Elegido 2015; Ferguson et al. 2011). In case of a natural disaster such as a flood, the sudden and simultaneous effects of increased demand and scarcity drive prices up which will deteriorate the condition of the flood victims. The result is that water, food, and shelter suddenly become unaffordable for people who are already struggling to cope with the disaster. Laissez-faire market advocates will typically defend such practices because they reflect free choices. Even if exorbitant, the higher prices will give suppliers an incentive to produce more of the needed goods. Therefore, the higher prices serve a social purpose and will tend to do more good than harm. The welfarists among the market advocates will argue that even if high prices will prompt a greater supply of goods, this benefit has to be weighed against the burden such prices impose on society as a whole, including those least able to afford them. On the other hand, the market moralists will point to the fact that in the wake of a national disaster buyers under duress have no freedom. In those circumstances, people have no choice but to purchase necessities like water and food and the exorbitant prices look more like extortion. The argument from corruption points out that even if price gouging stimulates entrepreneurial initiatives, excessive greed is a vice that a good society should discourage. By outlawing price gouging, a society affirms the civic virtue of shared sacrifice for the common good.

In this paper, we want to focus on the market moralists, who all argue for one reason or another that certain moral limits should be set to the use of the market mechanism. This argument has been made both by business ethicists, such as Santos and Laczniak (2009) and Sison and Fontrodona (2010); by economists such as Hirsch (1976) and Frey (1997); by legal scholars such as Ackerman and Heinzerling (2004) and Radin (1996); and in particular also by a group of political philosophers such as Anderson (1993), Grant (2012), Sandel (2012), Satz (2010), Stein (2001), and Walzer (1983). Some of these authors self-identify as members of the group by employing the very label MLM (Anderson, Sandel, and Satz); the others qualify on the basis of a theoretical criterion, that is, they all argue for some sort of divide between appropriate and inappropriate instances of market-based allocation (Ackerman and Heinzerling, Frey, Grant, Hirsch, Radin, Santos and Laczniak, Sison, Stein, and Walzer).

The main failure of the market moralists is that their arguments often seem arbitrary—each category seems to be argued for on a case-by-case basis, and there is no systemic solution which could be worked up into a unified theory of markets and morality. While some MLM theorists display more clearly than others, the ambition to develop a ‘more general theory for assessing markets’ (Satz 2010, 6), all market moralists can be seen to be involved in such a project to a greater or lesser degree (cf. Anderson 1993, 219–220; Radin 1996, 116–118; Sandel 2012, 11–15). But so far, each theorist is pursuing his or her own project and no unified theory of markets and morality seems to be in sight. We observe that after Walzer (1983) and Anderson (1993) introduced and further developed the idea of sphere differentiation, *none* of the later market moralists distinguishes properly between social domains, societal values, and allocation methods, three elements in the new conceptual framework that we introduce in this paper. Our paper therefore reconceptualizes the MLM debate by framing the issue of the market’s proper scope in terms of these three constructs. We suggest that in order to achieve a more thorough understanding of the allocation of goods and services, the debate needs to be conducted primarily in terms of values and domains, and not merely in terms of allocation methods.

We first review the MLM literature. We then introduce our new framework for analyzing the problem of MLM. To that end, we look at the social domain construct as it was originally introduced by Weber (1915), and was further elaborated upon by Walzer (1983) and Anderson (1993). Drawing on value theory (Rescher 1969; Schroeder 2013), we then argue that separate social domains will be characterized by their own configuration of values. Some of these values will play a role in more than one social domain, but each domain may be found to have its own characteristic configuration of values. Building on earlier work on allocative institutions, in particular by Calabresi and Bobbitt (1978) and Elster (1992), we further argue that particular allocation methods are incompatible with the realization of particular individual values. After introducing our framework, we consider education and healthcare as two sample domains from which the new conceptual scheme can be illustrated.

Before proceeding, we make a brief clarification about our scope and aim here. This article reviews and builds on the MLM literature, but it does not itself seek to develop and substantiate moral principles, as was done for example by Sandel (2012), Satz (2010) and Walzer (1983). Our overall goal in this paper is to provide methodological reinforcement for the market moralists so as to generalize the various strategies proposed by these authors.

## The Moral Limits of Markets Debate

### Origins

To reconstruct the MLM debate from its very beginnings, we would need to go back to classical political economists such as Smith, Ricardo, and Marx. These authors share a number of ideas about the nature and limits of markets which differ significantly from later ideas about the functioning of markets. First, classical political economists were well aware of the social embeddedness of markets. Second, they discerned that markets shape both individuals and societies as much as individuals and societies shape markets. For example, classical political economists already saw that the way the labor market is organized in a society will have an impact on the structure of public life in that society and shape workers’ capacities and preferences (Deane 1978).

But then, in the course of the 1870s, there was a relatively sudden paradigm shift, fueled by the work of authors such as Jevons (1871), Menger (1871), and Walras (1874). Owing to their ‘marginal revolution,’ the focus of economic analysis shifted to the question of how to optimize consumer preferences. The neo-classical paradigm took these preferences as given inputs so as to build a theory of market price. This forms the background for a position in modern economics, sometimes labeled ‘universal commodification’ (e.g., Radin 1996, 2–6). According to this position, provided that certain basic conditions are fulfilled, for example the prevention of theft and fraud, in principle any good or service will be most efficiently distributed by the market. In short, according to neo-classical economists, all goods are economic goods. Among the economists who have famously defended this extended use of the market are Arrow (1973), Becker (1976), Friedman (1962), Hayek (1960), and Posner (1977). For example, Becker did not shy away from applying his economic analysis to explain marriage and divorce (1976, 10, quoted in Sandel 2012, 50). Similarly, Posner concludes that ‘[r]ape bypasses the market in sexual relations (marital or otherwise) … and therefore should be forbidden’ (1977, quoted in Radin 1996, 86). It is against this idea of universal commodification that market moralists typically lash out.

### Parameters

In recent years, two central issues have emerged in the MLM debate. Market advocates claim that—market failure aside—the market will always provide the best distribution of all goods and services. Second, they reason from the (tacit) assumption that all goods and services can be commodified without affecting them. These two stipulations prompt two principal objections raised by MLM theorists against the market advocates:

- Objection 1: Markets may produce unjust results.
- Objection 2: Markets can degrade the value of a good.

An early example of this second objection was the study by the British sociologist Titmuss (Titmuss 1971) who argued that a market in blood changes the social understanding of blood donation from a ‘gift of life’ to a mere commodity. Indeed, by putting a price tag on all the good things in life we arguably open the door to a perversion of these goods. This happens in two ways. First, it may generate a type of self-contradiction. For example, friendship cannot be bought; purchased companionship—as a member of the leisure class may purchase the company of a valet or a ‘social secretary’—is self-defeating, or at any rate it is not the same as true friendship.^1^ Second, the market does not only distribute goods but also expresses a certain appreciation of those same goods. Economists typically assume that markets will distribute goods in a neutral fashion—that is, that the use of the market mechanism does not affect the goods thus traded. But in many cases this is not true. If we decide that certain goods can be bought and sold, we claim that it is correct to view them as merchandise. However, not all goods are can be valued meaningfully in the open market, and this is why, for instance, the buying and selling of people in slave markets was abolished (Radin 1996, 156–159; Sandel 2012, 9–10). For similar reasons you cannot buy or sell Nobel Prizes, or the right to vote in a democracy. Nobel prizes are intended to convey scientific accomplishment (*Ibid.*, 94). Civil rights and their correlative duties are not private property, but public responsibilities. To outsource them would be to degrade them (Anderson 1993, 158–159).

In order to complete a theory of MLM, market moralists therefore must address two problems:

- Problem 1: Which goods are suitable for market distribution and which are not?
- Problem 2: For those unsuited for the market, how they ought to be allocated?

We believe the current market moralists have failed to come to agreement on the former problem, and have failed to adequately address the latter. Yet, these two questions are critical. For anyone who accepts that the market has benefits to offer, but that within the market instances of distributive injustice likely exist (e.g., rich people having better access to privatized medical care), and that instances of goods being degraded likely exist (e.g., the purchasing of university degrees), our two questions become most salient.

### The Market Moralists

The market moralists can trace their roots back to Walzer (1983). In direct contrast to the neo-classical economists, the core of Walzer’s argument was that all goods must be considered social goods and that criteria for the distribution of these goods should be derived from their social meaning. On that basis he distinguished eleven distinct societal ‘spheres’ such as economics, politics, security and welfare, and religion. Walzer argued that each sphere will be characterized by its own standards of justice. Consider these examples: public office is by definition public; the goal of punishment is to either condemn particular acts, deter others, or reform perpetrators; divine grace is a gift of a gracious God. Thus, goods such as political office, criminal justice, and divine grace by their very nature ought not to be for sale (Walzer 1983).

Some have argued that Walzer’s actual division of spheres is too ad hoc (e.g., Anderson 1993, 143; Néron 2010, 338; Radin 1996, 46–49; Satz 2010, 81). We will suggest in this paper, however, that while the number and nature of the spheres may be contestable, it does not negate the concept of there being different domains of a society in which different values may obtain. But it does seem that his argument regarding the suitability of allowing money into the spheres beyond the economic was too ad hoc. Too often his work relies on uncritically evaluated social conventions. In what sense does money ‘belong’ to the economic sphere such that it is inappropriate, even immoral, for it to be used in other spheres? And if Walzer holds that we should take into account the social meaning of a good, this hardly helps us allocate a good like public office. By itself, the idea of spheres does not tell us how to distribute a particular good.

Two market moralists coming in the wake of Walzer, Anderson and Radin, have therefore attempted to improve on his conventional, and somewhat uncritical, notions of what constitutes justice in the varying spheres of society. Anderson’s work was occasioned by the increasing popularity of ‘economistic political theories’ (1993, xii) starting from Downs (1957). She objected to the hyper-rational agent at the center of these theories, opposing such ‘a socially impoverished conception of the individual’ (1993, xii) and how it results in a far too simplistic view of the process by which people come to their choices. These economic theories of politics reduce human conduct to a single mode of valuation, namely the instrumental use of a good, ignoring the many other modes of valuation by which people can actually respond to a good such as ‘love, admiration, honor, respect, affection, and awe’ (*Ibid.*, xiii). By tracking differences in the ways we appropriately value-specific goods, we can determine which goods are properly treated as market commodities.

Anderson concludes that policy issues such as surrogate motherhood, privatization of public services, and environmental protection should not be viewed as tradable commodities, but as objects of respect or reverence. From these particular instances she generalizes outwards, identifying five features characteristic of market relations. Goods can be appropriately distributed via the market if they are impersonal, egoistic, exclusive, want-regarding, and oriented to ‘exit’ rather than ‘voice’ (1983, 145).

Radin (1996) seeks to develop a criterion for whether markets are appropriate primarily based on Kantian respect for persons. She argues there are some goods that are ‘identifiably self-constitutive’ (1996, 49) and important to personhood and the flourishing of the individual. Examples of such ‘internal’ goods (*Ibid*., 52) are a woman’s sexuality and reproductive capacities. These goods should not be distributed through the market, e.g., via prostitution and paid surrogacy, because commodification is harmful to the personhood of those involved (*Ibid.*, 49–58).

Radin’s attempt to formulate a divide between appropriate and inappropriate uses of the market is complicated by her introduction of the possibility of partial commodification. For example, in many farming communities children may work alongside their parents in the fields at harvest time. Ultimately, then, it is left unclear which goods may be commodified, which may be partially commodified, which not commodified at all, and in the event of the latter, how they ought to be allocated. The two problems of the market moralists thus go unanswered.

The author to have contributed most to actual theory building on the problem of MLM was Satz (2010).^2^ She identified four basic parameters for assessing markets. Markets suffer from vulnerability if ‘some people are so poor or so desperate that they accept any terms of exchange that are offered’ (2010, 9). Markets have weak agency if information asymmetries put some participants at a disadvantage and thus prevent them from looking out for their welfare. Markets produce harmful outcomes for individuals if they ‘become destitute or … their most basic interests are undermined.’ And markets produce harmful outcomes for society if they ‘undermine the framework for a society of equals,’ or ‘support relations of humiliating subordination or unaccountable power’ (2010, 9). If a market scores poorly in regard to one or more of these criteria, she deems it ‘noxious.’

Satz applies her criteria among others to the market for kidney transplants. She observes that a noxious black market for kidneys has arisen in poor countries. In such countries, the extreme poverty of people makes them vulnerable, and their inadequate levels of education create the information asymmetry that typifies weak agency. The sale of kidneys often produces harmful outcomes for the individuals selling kidneys due to the lack of medical attention they receive after the operation, and highlights the division between the haves and have nots. Thus in poor countries, the market for kidneys fails on all four criteria.

But even if Satz’s four criteria can do the heavy lifting she has designed them to do, they would be insufficient when it comes to the second problem of the market moralists—how to distribute goods if they are unsuited for the market?

The latest contribution to the MLM debate to date is Sandel (2012).^3^ He notes the two principal objections of the market moralists—first, that allocation via the market may lead to injustice; and second, that markets will in some cases corrupt the goods they allocate. In addition, Sandel also makes three descriptive claims with respect to the expansion of the market at the cost of other areas. He first argues that the introduction of the market mechanism has been so gradual as to have gone hardly noticed. He further observes that precisely because the introduction of the market mechanism was so gradual, there never was a principled discussion about the propriety of this transition from conventional norms to market norms, and thus he calls for public debate. Third, he asserts that markets crowd out non-market mechanisms. However, he gives no further explanation how this happens.^4^ But he does not work out any operationalized criteria that could help distinguish between appropriate and inappropriate usage of markets.

The strength of Sandel’s work, therefore, may lie in problem description rather than in problem solution. His many colorful examples, though eclectic and unhelpful in guiding his readers toward a solution, have served to re-ignite the debate. They have convinced many that the public debate he calls for—to question the appropriateness of the market for the distribution of many goods—is indeed badly needed.

In our paper, we seek to provide some insight into both of the problems market moralists must address: What goods are suitable for market distribution? And if not suitable, by what alternative method ought they be distributed? In doing so, we will draw upon the concept of domains that Walzer introduced and Anderson built on, upon values that Anderson explicitly introduced to the debate, and advocate the broadening of the debate beyond academics to involve more directly the public as first argued by Stein, and elaborated upon by Sandel. We will add to the mix a discussion of allocative institutions, broadening it beyond the simple dichotomy—market versus non-market allocation—that market moralists currently focus on; provide a structure that relates domains, values, and distributive methods to one another; and finally provide some focus to the public debate that needs to occur. We will start our analysis by introducing the three basic concepts of our new conceptual scheme.

## Domains, Values, and Allocation Methods

### The Domains of Society

Max Weber was among the first to view society as being composed of different domains—such as the commercial, the personal, the political, the romantic, and the spiritual domains, in which different values may be argued to predominate. It was Weber’s contention that individuals bring meaning to their lives by orienting themselves around one single value, and that the values they choose from among are potentially conflictual (Weber 1915). Weber’s conceptualizing of the various social domains may, in turn, have ultimately been related to the distinction between a public and private sphere in ancient Greek philosophy. In the world of the ancient Greeks, participation in politics (the public sphere of the polis) assumed ownership of a private household of women, servants, and slaves to do the work. For Plato and Aristotle, it was natural that all citizens had their own well-run private household, to provide for their material needs, so that they could afford to devote their attention to public matters. Hannah Arendt has shown how this originally crisp and clear distinction was decisively altered by the rise of ‘the social’ domain (1958, 38–50).

Following Weber, this approach was elaborated by the German social psychologist Eduard Spranger (1928), who outlined six domains, and later taken up by the market moralists Walzer (1983)^5^ and Anderson (1993). Walzer’s (1983) inquiry into a set of distinct ‘spheres of justice’ was intended as a rejoinder to the Kantian universalism of Rawls’ (1971) original project. The idea of a universal theory of justice does not take sufficient account of the different social spheres in which people interact. Walzer believed that what constitutes justice in a particular case will depend on the social sphere with which one is dealing, and each has to be associated with its own criteria for justice. Similarly, Anderson, claims that goods differ in kind because they are valued according to different ‘modes of evaluation’ that vary from one societal domain to another. For example, goods with only a use value, such as a hammer—desired solely because it helps us achieve ends—belongs in the commercial domain. However, education, which she argues has an intrinsic value in addition to a use value, does not belong in the commercial domain (1993, 199–201).

The key point of the domain theorists is that they advocate a pluralist view of society. They argue that each domain contains different values, and the values are not reducible to a single, all-encompassing one. Therefore, they differ in approach, for example, from the utilitarians who favor welfare or happiness as overriding values. The number of values Anderson chooses to focus on, though, is quite limited—she references only fraternity and democratic freedom (1993, 158–163). Walzer touches on a far wider range of values, but limits his discussion of each to particular domains. As examples, equal opportunity is discussed only as it pertains to seeking office in the political domain (and not for instance as it pertains to careers outside that domain), and freedom shows up only in isolated discussions regarding marriage, academics, and voting (1983, 131–132, 235–238, 284, 317–318); efficiency is not discussed at all.

We believe the approach of these theorists, which begins to link domains and values, is insightful and wish to pick up where they left off. Our intention is to argue that the different values will point to different allocative methods, thus linking the allocative methods back to domains. Table 1 systematizes the results of these four earlier versions of domain differentiation. As much as possible we have used the authors’ original terminology, categorized the distinctions made by the authors themselves, and rendered them in alphabetical order.

**Table 1:** Various proposals for domain differentiation

Domain	Weber (1915) (section #)	Spranger (1928)	Walzer (1983) (chapter #)	Anderson (1993) (chapter #)
1. Aesthetic	Aesthetic (6)	Aesthetic		Art (7.3)
2. Economic	Economic (4)	Economic	Money & commodities (4)	Market
3. Education & science	Intellectual (8)	Theoretical	Education (8)	Science (7.3)
4. Environment				Environment (9)
5. Health			Security and welfare (3)	Surrogacy motherhood (8)
6. Leisure			Free time (7)	Clubs
7. Politics	Political (5)	Political	Political power (12)	State (7.5)
8. Professions			Office (5), work (6)	Professions (7.3)
9. Relationships	Erotic (7)	Social	Kinship & love (9)	Associations, family, friendship (7.4)
10. Spiritual	Moral and religious (9)	Religion	Divine grace (10)	Religion

For the purposes of this paper, we need not reconcile the differences among the theorists, nor formulate an argument regarding which list of domains is most correct, nor even necessarily defend our interpretation of the scholars’ work and the domains we associate with them. Here we only wish to recognize that different domains have been distinguished within a society by at least four prominent exponents of the idea of domain differentiation. We also observe that none of these authors explicitly links their discussion of domains to specific values or allocation methods. We will now proceed to introduce these two additional theoretical apparatuses.

### Values and Allocation Methods Defined and Illustrated

There is a rich and subtle philosophical literature on what constitutes a value (e.g., Gaus 1990; Hsieh 2008; Nagel 1986; Parfit 2011; Putnam, 1981, 2002; Raz 1986, 1999; Scanlon 1999; Sen 1988). For present purposes, we will summarize this literature by defining values as reasons for action, which guide people’s choices and enter into their deliberations. In addition to this general philosophical literature, there is also a vast methodological literature on the role of values in science and how value claims differ from factual assessments (e.g., Douglas 2011; Hempel 1965; Kuhn 1977; Nagel 1961; Weber 1949). Values moreover vary according to the level of analysis one uses (Agle and Caldwell 1999). They may be macro-level in nature, such as cultural (Hofstede 1980; Kirkman et al. 2006); or micro-level in nature, for instance at the personal level and specific to managers (e.g., Bass and Bass 2008; Cressey and Moore 1983). The values we are interested in are meso-level in scope, and are the ones that have historically been used as the basis for the distribution of goods and services within societies. Examples of these societal values include beauty, convenience, efficiency, equality, freedom, loyalty, merit, need, truth, and welfare (e.g., Frederick and Weber 1987; Martin 1981; Pearson and Chatterjee 2001; Rokeach 1973; Schwartz 1994, 1999).

As for the classification of different methods of allocation, the analysis in this paper goes back to the idea of ‘pure allocation approaches’ proposed by Calabresi and Bobbitt (1978). Following Elster (1992), we will define allocation methods here as the social institutions by which scarce goods and necessary burdens are allocated. In Table 2, we include a partial list of allocative methods and their associated societal values.

**Table 2:** Societal values and allocation methods

	Allocation methods				
	Attributional assessment	Free market	Lottery	Queue	Rationing
Values					
Efficiency		✓			
Equality			✓	✓	✓
Freedom		✓			
Merit	✓				
Need	✓				✓
Status	✓	✓			

Our goal in presenting this table is not to produce an exhaustive matrix of every value and allocation method, but merely to give the reader a flavor of the interplay between the two concepts. With that in mind, then, we will consider the various entries in those cells. Efficiency is conceived and operationalized in different ways. Economists usually think about efficiency in terms of optimizing individual preference satisfaction, commonly known as Pareto-optimality. From this perspective, a distribution is efficient if nothing can be changed, without diminishing the preference satisfaction of at least one other individual. In his own writing, Pareto distinguished between ‘ophemality,’ referring to the satisfaction people get from economic goods, and ‘utility’ which stands for a broader notion of satisfaction derived not only from goods but from social and political institutions as well. In addition to these two traditional meanings of efficiency, some scholars still distinguish dynamic efficiency, which roughly coincides with the innovation capacity of an economic system (Nooteboom 2014). In this paper, we proceed from a more casual idea of efficiency as maximal output for a given input. Vice versa, the idea of realizing identical goals at lower costs—thereby preventing waste—also carries the positive connotation people associate with efficiency (cf. Ackerman and Heinzerling 2004; Stein 2001). This appraisive use of the concept of efficiency is consistent with the definition used by authors such as Heath (2001). The most common allocation method used to promote efficiency in this sense is the market. The free market does well at squeezing out inefficient producers, because *ceteris paribus* rational consumers will choose the lowest priced option. Thus, those manufacturers with the more inefficient production operations—that is, with costs higher than their competitors—will be unable to get their selling price down low enough to attract consumers (while still covering their expenses), and will therefore go out of business. And also in alternative interpretations of the market mechanism, such as the evolutionary perspective suggested by Sugden (1986), efficiency is naturally associated with markets.

Equality can be furthered by at least three allocation methods: lotteries, rationing, and queuing. The queue is likely to be used to allocate a somewhat scarce, but not critical good or service, such as a cashiers’ time and attention at supermarket check-outs. Lotteries and rationing are used for more critical goods. A lottery guarantees an equal probability of obtaining a good that exists in discrete non-divisible units, such as kidneys (Waring 2004), while rationing guarantees an equal outcome (i.e., allocation) for a good that exists in bulk such as flour (Cornes and Sandler 1996).

The societal value of freedom is commonly interpreted in terms of either or both of two major forms, designated with the labels negative and positive (Berlin 1969). Negative freedom entails no physical interference from others and laws primarily being put in place to prevent such interference. One allocation method for promoting negative freedom is the free market, because broadly characterized, in a setting free from physical interference, getting those goods and services one wants depends on one’s ability to offer up something of value—tangibles such as product or money, or intangibles such as one’s labor. Both buyers and sellers choose their best offer, and the interplay of demand and supply sets prices free of the undue bias of any individual or group policy. This setting of maximal negative freedom is, of course, the libertarian conception of the free market (Nozick 1974). Positive freedom, on the other hand, may roughly be operationalized as self-realization. It refers to the possibility of developing one’s talents and capabilities (Sen 2009), and enabling others to realize the potential they have in them. In its positive meaning, freedom will be realized by attributional assessment, where an individual is allocated a good based on the presence or quantity of some personal characteristic (Wempe 2004). In the context of positive freedom, parents may seek to enable their children, by looking at talents and qualities they appear to possess, and then trying to develop those.

Merit is most likely promoted by an attributional assessment—a performance review evaluating a person’s previous accomplishments. For instance, a key attribute in determining access to grant funding, in the case of university professors, may be the number of publications in top-tier journals. Key attributes considered for access to the best graduate educational programs, in the case of university students, may be the quality of their undergraduate program, their grade point average, and the stature of the people writing their recommendation letters.

The fifth value, need, may also be promoted by means of attributional assessment. Triage, where people are assessed in terms of a particular attribute—the urgency of their medical condition—determines access to the services of a hospital emergency room. At other times attributional assessment may require a formal appraisal of a demographic variable. For instance, the attribute evaluated for access to an elementary school free lunch program would be parental income (Levine 2008). For scarce goods that are considered essential to everyone’s survival, need may also be advanced by rationing. In 1940, for example, rationing was used to meet people’s needs for basic caloric intake in England during the Second World War, when it was estimated the nation only had food stocks sufficient to last 6 weeks (Elster 1992). Therefore, as a societal value, need (relief) is advanced by at least two allocation methods—attribution and rationing.

By status we mean wealth, and to a much lesser degree pedigree. Leading the privileged life, something so many people aspire toward, traditionally required the possession of a particular attribute—specifically, pedigree. Admission into some social or country clubs is still at least partly based on the presence of one’s last name in some social registry, and one’s ability to demonstrate appropriate lineage to that name (Domhoff 1974; Higley 1995; Sherwood 2012; Veblen 1899). However, in America, along with some other parts of the world, the life of status is also promoted through the free market. In the marketplace, one purchases the goods and services of elite schools and resorts, and access into exclusive gated communities and nightclubs. Thus similar to equality and need, we see status as a value being realized by at least two allocation methods—in this case attribution and the free market.

### The Interplay of Domains, Values, and Allocation Methods

In this section, we examine the relationships between the three variables of our conceptual framework. Values form the backbone of this framework, and so we will discuss values and their relationship to allocation methods, and then discuss values and their relationship to domains.

#### Values and Allocation Methods

We note again here that Table 2 is not meant to be an exhaustive survey of societal values and allocative methods. Rather, it is designed to make evident the following observations regarding the relationship between values and allocation methods. At least three obvious results can be seen:

1. Allocation methods support some values, but not others. A lottery, for example, is commonly taken as an allocation method that supports equality—it is used to assure everyone having an equal probability of obtaining an outcome. It would be hard to imagine lotteries as an allocation method that would promote a value like merit.
2. Allocation methods can support more than one value. For example, attributional assessments may be used to support merit, and need, as well as status. It will support merit as in the case of a performance review where a granting agency may review a scholar’s CV in search of a particular attribute, such as publications in top-tier journals. It will support need, as in the triage performed at the emergency rooms where hospital staff seek to identify a particular attribute of an individual—the urgency of their medical condition. Or it may support status, as in a background check completed by an exclusive country club in search of a particular attribute, such as ‘proper’ lineage. Thus, attributional assessments—be they in the form of performance reviews, triage, or background legacy checks—may be used in order to promote multiple values. As to the market mechanism, it is widely acknowledged that markets will support efficiency (e.g., Buchanan 1985, 14; Okun 1975, 50–51; Satz 2010, 17–21), freedom (e.g., Buchanan 1985, 78; Nozick 1974; Satz 2010, 21–26), and status (e.g., Buchanan 1985, 81-87; Sandel 2012, 172, 201).
3. Values can be supported by more than one allocation method. For instance, the value ‘equality’ can be promoted by lotteries, rationing, or queues (Fleurbaey 2007, 2008). Similarly, need can be promoted with at least two different allocative methods—attributional assessment (Daniels 1985, 26–28) and in some cases rationing (Daniels 1985, 14–15).

#### Values and Domains

We can make some similar observations regarding the number of values and number of domains and how they correspond to one another.

1. Some domains have more than one value. Sometimes these values co-exist on an equal footing, sometimes one has priority over the others. Healthcare, for example, has at least two values associated with it. As was shown by Daniels, need typically acts as the dominant value, and equality as a subordinate value. Daniels (2013) specifies two main ‘conceptualization[s] of equitable access to healthcare among health service researchers,’ that is, ‘the use-per-need view’ and ‘the modified market view.’ The use-per-need view is supported by scholars such as Aday (1975, 2001); Aday and Anderson (1974, 1975); and Aday et al. (1980) and proceeds from ‘the idea that the utilization of services should reflect actual needs for care.’ The modified market view, which is among others expounded by Enthoven (1980), ‘focuses on the availability in the market of a decent basic minimum of care’ (all healthcare sources quoted in Daniels 2013). Both these main conceptions build on the idea of healthcare needs as a basis for a theory of the just distribution of healthcare services.
  Daniels (1985) concludes ‘we must talk about healthcare *needs* if we are to explain what is special about healthcare and thus be in a position to give an account of distributive justice for it’ (1985, 23, emphasis in original). But because the concept of needs has been ‘in philosophical disrepute,’ being ‘both too weak and too strong to get us very far toward a theory of distributive justice,’ it must be combined with some notion of ‘fair equal access.’ On the one hand, the concept of needs is ‘opportunistic’ in that we tend to refer to the means necessary to reach any of our goals as ‘needs.’ On the other hand, the idea of ‘needs’ distinguishes insufficiently between what is basic and what is not-basic.
  The prominence of need and equality as a subordinate value in the healthcare domain can also be seen from the familiar practice of triage (Sirgy et al. 2011). For example, in the waiting area of an emergency room, specially trained nurses will quickly examine patients, and sort them into rough groups. The group with the most acute and life-threatening conditions will then receive the first attention. In any given group, patients will be viewed as equals and the order of arrival to the emergency room is most likely used to determine who gets the first attention. Thus, triage is used to divide people into groups, and to establish priority between groups, and then queuing is used to establish priority within groups.
2. Some values exist in more than one domain. Need-based allocation plays a role in both the healthcare domain and the education domain. The former—involving triage—has already been discussed. Regarding a similar combination of need and equality^6^ in the educational domain Walzer points out that ‘educational equality [can be seen] as a form of welfare provision, where all children, conceived as future citizens, have the same need to know, and where the ideal of membership is best served if they are all taught the same things’ (1983, 203). Need has also been used to justify the allocation of hot lunches at elementary schools, as well as need-based scholarships to students and subsidies to schools (e.g., Feinberg 1998; McPherson and Schapiro 1998; Newman et al. 2010; Posselt 2009).

## Illustration from Two Sample Domains

Having outlined the relationship between domains, values, and allocation methods, we can now translate the MLM debate into terms of the conceptual scheme developed here. In order to focus our discussion and to help make it a more concrete, we apply our conceptual framework once again to the two sample domains of healthcare and education and some relevant issues pertaining to them. We believe that focusing the discussion on values will make it clear which allocation methods are permissible for use in a domain, and this should thus help make clear why certain actions would be inappropriate.

### Healthcare

In regard to the healthcare domain, one example prominent in the debate is the sale of body organs, specifically kidneys.^7^ In most countries, people may donate their kidneys, but not sell them on the open market. But some disagree with this regime, and argue for a free market for kidneys (e.g., Cherry 2005; Hippen 2005; Taylor 2005). On the one hand, the argument is that thousands of people die each year waiting for kidney transplants. On the other hand, people in need of money should be free to sell their kidneys if they wish.

The core argument for permitting the buying and selling of kidneys rests on the libertarian notion of self-ownership, that is, if I own my body, I should also be free to sell my body parts as I please. But most proponents of markets in kidneys stop short of embracing the full libertarian logic, as Sandel (2010) shows on the basis of two thought experiments that isolate the pure element of self-ownership. The first case is an eccentric art dealer who sells human organs to affluent clients as coffee table conversation pieces. The second case concerns a subsistence farmer in an Indian village who has already before sold his first kidney in order to raise money to educate his first child. When his second child approaches college age, and another buyer offers a handsome price for his second kidney, should he be free to sell his second kidney too, even if that would kill him? Sandel concludes that ‘[i]f the moral case for organ sales rests on the notion of self-ownership, the answer must be yes. It would be odd to think that the farmer owns one of his kidneys but not the other…. if we own our bodies and lives, then the farmer has every right to sell his second kidney, even if this amounts to selling his life’ (2010, 72).

If all this constitutes an adequate assessment of the values appropriate to the healthcare domain, the implication of applying our new conceptual scheme would be that the sale of body organs in the market will lead to an allocation not in accordance with the values of that domain. The neediest may not receive the organs; rather we would expect the privileged to. If need and equality are to persist as values in healthcare, then an attributional assessment must be performed to determine who is in most need of a kidney, with perhaps a lottery used to determine who is to be served first among those of equal need. Other subordinate values may come into play, for example, some sense of effectiveness may be important—if a patient is too close to death, transplanting a kidney may be ineffective. The point here is that certain allocation methods (attributional assessment, lottery) become appropriate given the values in play, and other allocation methods become inappropriate (market, queuing).

As far as business participation is concerned, in what can clearly be a very complicated public policy-making process, companies would need to focus more directly on values appropriate to a societal domain, independent of their traditional claim to enhancing efficiency. In the domain of healthcare, it would mean that while there is nothing wrong with trying to make processes more efficient, this should not be achieved at the cost of the primary values of the healthcare field, such as satisfying the needs of the patients (c.f., Stein 2001; Weber 2001). Similarly, various approaches taken by businesses for underwriting costs in the healthcare field need to be brought under the lens. For example, consider concerns that exist regarding the role that pharmaceutical company representatives play in the provisioning of drugs to the medical field. Pharmaceutical companies heavily subsidize many conferences for doctors (Relman 2001; Rodwin 2013), and in so doing have access to the doctors and more importantly may create a sense of indebtedness—such that doctors feel obliged, almost unconsciously, to prefer the drugs of the pharmaceuticals providing them with perquisites (Sah and Fugh-Berman 2013; Vashi and Lakowski 2012). Such commercial sponsorship in healthcare should not be judged exclusively in terms of the financial assistance it provides, but rather with a view to how well such efforts go together with the primary aims of healthcare. Administrators in the healthcare domain, while they are under pressure from diminishing funding by governments or insurance providers, would do well to take account of how this may corrupt the values associated with their domain.

### Education

In regard to the education domain, consider developmental admissions. While many readers are familiar with legacy admissions (giving an edge in admissions to the children of alumni), less familiar to readers and probably more insidious are ‘development admits.’ At many universities, there are ‘applicants who are not children of alumni but who have wealthy parents able to make a sizable financial contribution to the school. Many universities admit such students even if their grades and test scores are not as high as would otherwise be required’ (Sandel 2010, 182). However, in the educational domain merit has long been a cornerstone value. Attributional assessment is a provision mechanism that can promote this value, while the market cannot. Developmental admissions will produce a skewed allocation of admissions—favoring the privileged class—giving some groups in society an unjust advantage. Additionally, these kinds of admissions will have a corrupting influence on the educational good itself. Opening the door to making education a purchasable commodity corrupts it, because a degree no longer represents knowledge acquired solely through merit, but makes it instead a credential partially purchased with one’s wealth. Therefore, assuming merit is to remain as a central value in education, purchasing admission is inappropriate, and must not be engaged in, even if it provides funds for the university.

It follows, then, that the call for a public debate made by various MLM scholars (Sandel 2012; Satz 2010; Stein 2001) must be conducted in regard to *values* such as efficiency, freedom, and equality, rather than in regard to *allocative methods* such as the market or the queue. Do people value efficiency or freedom so much that they are willing to bring the market mechanism into other domains and run the risk of it subordinating the pre-existing values (such as need, equality, and merit) of those domains? Or consider a variation of this question in the context of education, where equality emerged as a key value in America after the Second World War (Ehrenberg 2006). Starting in the 1950s, numerous educational programs assuring equality in education were developed. For example, remedial instruction for the needy, or special education for the differently abled, has been implemented at great cost to the members of society. However, if we are now less willing to fund such costly programs, such that administrators feel obliged to introduce aspects of the market mechanism into their school—say through developmental admits—the administrators will necessarily open the door to privilege, thus putting at risk the very value (equality) they were trying to promote. Those responsible for administering a domain—in this case educational administrators—thus struggle to manage the mixed message society sends them (“Yes, equality is important and so you must provide special educational programs, but no, we are unwilling to fund them to the necessary extent.”) Perhaps, then, it is time to bring the point to the public forum for a debate: Is equality to remain a value in the educational domain or not? (And for the answer to be truly “yes,” then the funding must be there to support it.) In other words, debate is needed and it must be more about values than allocative methods. Within the broad public forum of our society, we need to decide which values are important to a domain, and then having done so it should be more apparent which allocative methods should or should not be used in that domain.

Such debates regarding the values appropriate to a domain may need to be reopened periodically—since societies are dynamic in nature—and thus it should be understood by all that questions regarding values will never actually be permanently settled. Additionally, it should be clear that value changes and resulting changes in the use of mechanisms will often be controversial, though not necessarily so. One example of this is England during WWII. Originally, gas was allocated via the free market, for as with many commodities, negative freedom is often an important value—that is, a prevailing view in Western society is that people should be free to trade as they please, unless it interferes with other people’s ability to do so. However, upon the outbreak of war, supplies from the Persian Gulf were threatened, and simultaneously the British government began stockpiling fuel. A shortage occurred, and in the name of equity, the Government began to ration petrol, trying to make sure everyone had access to the fuel they needed, even if in reduced amounts. The Government’s decision to adopt a new value (equity) and distribution mechanism (rationing) proved highly controversial, as Britain’s survival was not yet in question. Once the Battle of Britain began, though, the sale of petrol was prohibited. It could be used freely for those efforts deemed critical to Britain’s survival, such as for running farm machinery; otherwise its use was forbidden. It was free, because having to pay for it would have put users out of business—it was so valuable. In other words, it had become literally priceless (Zweiniger-Bargielowska 2000)—not just invaluable but actually un-valuable. This time the Government’s decision to adopt a new value (need) and distribution mechanism (honor system) was non-controversial—the public patriotically accepted this decision.

Lastly, this discussion raises the question of why the market is intruding into these other spheres in the first place, as market moralists claim (Anderson 1993, 141; Radin 1996, 95–101; Sandel 2012, 6–8, 93–130; Satz 2010, 3; Walzer 1983, 120–122). The domains of healthcare and education are both dealing with escalating costs (Daniels 1985; Newman et al. 2010; Nooteboom 2014). The market’s pricing mechanism is quite effective at squeezing out inefficient suppliers and pushing prices down, thus helping to make costs more manageable. Thus, in the case of education and healthcare, there are cost concerns and a desire to bring expenses under control, and this most likely accounts for the choice to introduce the market mechanism into these domains.

The tacit assumption that administrators in education and healthcare must be making is that the principal values of their domains can still be achieved, but more cheaply and more readily (and thus more efficiently) via the market mechanism. The reality, though, is that *if* society values need as a basis for administering healthcare and merit as a basis for administering education (for example), the market may be ill-suited as an allocation method for either of those domains, since it promotes values other than need and merit.

## Conclusion

### Implications for Theory

This paper builds on and seeks to extend the work of a group of theorists we have labeled market moralists, all of whom seek a solution to the MLM problem, that is, where to draw a line between goods and services that can appropriately be allocated by the market, and goods and services that are unsuited for market allocation. Current market moralists have put forward various proposals, but they can be shown to suffer from the following shortcomings. (1) All market moralists discussed here tend to conceptualize the MLM problem as a simple dichotomy of market vs. non-market allocation; (2) some tend to look too much for universal criteria for the distinction of particular ‘noxious’ markets or incentive systems (Grant, Sandel, and Satz); or (3) stage their arguments for particular ‘fungible’ goods on a case-by-case basis (Anderson, Radin, Sandel, Stein, and Walzer); (4) finally, all market moralists discussed fail to address the logical follow-up question: if goods are unsuited for the market, how should they be allocated?

This paper has sought to address these weaknesses. In our proposal, the problem statement for the MLM debate needs to be refined and sharpened by distinguishing different social domains. Accordingly, we re-introduce to the debate the concept of domain differentiation as already proposed by Walzer (1983) and Anderson (1993). However, we couple the distinction of social domains to values and allocation methods. We suggest that a characteristic (set of) value(s) can be associated with each separate domain, and in turn every value will be supported by an accompanying (set of) allocative method(s). While our configuration of values and allocation methods in Table 2 certainly is not intended as a complete and definitive typology, the table suffices to give a flavor of their interplay. Furthermore, it helps explain why an allocation method such as the market is ill-suited for certain domains. The market may promote values that are foreign to some domains; for example, the market benefits the privileged, and status is a value that is out of place in the healthcare domain. Our three-part conceptualization of domains, values, and allocation methods forms the foundation for a possible moral theory of markets; markets ought not be used as an allocative method where any of the values it necessarily promotes (e.g., negative freedom, efficiency, status) are not values of the domain in question. The three-part conceptualization should further be able to identify which allocation methods are permissible in a particular domain. A value such as equality in a domain may make permissible the use of rationing, for example assuming the good or service is divisible, provided rationing does not undermine another value in that domain. Thus, the theoretical contribution of this paper for the MLM debate may be summarized in terms of three propositions:

1. The discussion on the moral limits of markets cannot be conducted in categorical terms, but needs to differentiate between social domains.
2. Each of these social domains may be found to have its own values.
3. Different values will be realized by different allocation methods.

Only with these three propositions in mind will it be possible to have a productive debate on the use of the market, or for that matter any allocation method, in a given part of our society today.

### Implications for Research

This paper has only sought to indicate where the current market moralists go wrong and how their argument conceivably could be conducted in the future. The generalization of earlier proposals for domain differentiation (summarized in Table 1) was merely intended as a comparison of the four earlier domain theorists we reviewed and still needs to be refined and elaborated. In theorizing the idea of social domains, we should include in particular the political science and policy analysis literature (Dahl and Lindblom 1976; Laumann and Knoke 1987; Musgrave and Musgrave 1984; Weimer and Vining 1992; Wildavsky 1979) as well as the typology of institutional orders elaborated in the institutional logics perspective (Thornton et al. 2012). Together with the example of the configuration of societal values and allocative methods in Table 2, this could be the source of a major empirical research effort designed to test further examples both in healthcare and education and beyond these two sample domains exemplified in this paper. For example, managers in both the leisure domain (which according to some domain theorists could include the entertainment industry and sports) and the environmental domain face questions regarding the appropriateness of the market to those domains. To decide what should and what should not be for sale for money, then, we will need to determine which values in the different social domains we want to respect. In this regard, recent market moralists have done important work, but we need to go about these matters more systematically and methodologically than has been done so far. Another topic for future research along the lines sketched here is related to the fact that debates involving values will likely involve the problem of the essential contestability of value-concepts (Gallie 1956), a discussion with a long pedigree (e.g., Collier et al. 2006; Connolly 1983; Gaus 2000; Gray 1977; Swanton 1992), which is still ongoing.

## Footnotes

1 However, see Chang (2001) for an argument in support of the idea that friendship can be bought for money.

2 For reviews of Satz (2010), see also Brown (2014), Claassen (2012), and Maskivker (2009).

3 For reviews of Sandel (2012), see also Besley (2013), Brown (2014), and Chandler (2014).

4 One potential explanation is that markets ‘don’t pass judgment on preferences they satisfy. They don’t ask whether some ways of valuing goods are higher, or worthier than others’ (2012, 14).

5 With the benefit of hindsight, there is a clear connection to be seen between several of these authors anticipating the idea of domain differentiation. But, interestingly, Walzer only mentions Weber in regard to ‘patrimonial’ society and enforcement of religious holidays (1983, 129, 193), while Anderson cites Weber only in regard to action types and market ethics (1993, 22, 227); however, neither acknowledges his work in regard to domains.

6 The particular configuration of need as a dominant value and equality as a subordinate value constitutes an important parallel between the healthcare and education domains. *‘Both address needs which are not equally distributed among individuals’* (Daniels 1985, 46, emphasis in original).

7 This and the following paragraph build on Sandel (2010, 70–72). Parallel arguments are made by other market moralists, e.g., Radin (1996, 8, 21, 23–24, 48, 51, 124) and Satz (2010, 189–205).
